# Compared Morphology of the Immatures of Males of Two Urban Ant Species of *Camponotus*

**DOI:** 10.1673/031.012.5901

**Published:** 2012-05-02

**Authors:** Daniel Russ Solis, Eduardo Gonçalves Paterson Fox, Mônica Lanzoni Rossi, Odair Correa Bueno

**Affiliations:** ^1^ Centro de Estudos de lnsetos Sociais, lnstituto de Biociências, São Paulo State University (UNESP), Rio Claro, São Paulo, Brazil; ^2^ lnstituto de Biofísica Carlos Chagas Filho, Federal University of Rio de Janeiro (UFRJ), Rio de Janeiro, RJ, Brazil; ^3^ Laboratório de Histopatologia e Biologia Estrutural de Plantas, Centro de Energia Nuclear na Agricultura, University of São Paulo (USP), Piracicaba, São Paulo, Brazil

**Keywords:** Formicidae, Formicinae, *Myrmothrix*, *Tanaemyrmex*, larvae

## Abstract

The immatures of males of two species of *Camponotus* ants (Hymenoptera: Formicidae) are described and compared by light and electron microscopy. The numbers of larval instars were determined: *Camponotus rufipes* Fabricius (Hymenoptera: Formicidae) have four instars; and *Camponotus vittatus* Forel have three. Male larvae of the two species are similar to previously described *Camponotus* larvae, sharing the following traits: basic shape of body and mandible, presence of ‘chiloscleres’, ‘praesaepium’ (some specimens), labial pseudopalps, and ten pairs of spiracles. However, larvae of the two species can be separated by bodily dimensions and based on their hair number and types. Worker larvae of *C*. *vittatus* previously described are extensively similar to male larvae, with only a few inconspicuous differences that may result from intraspecific variation or sexual differences.

## Resumo

Os imaturos de machos de duas espécies do gênero *Camponotus* (Hymenoptera: Formicidae) foram descritas e comparadas, com o auxílio da microscopia óptica e eletrônica de varredura. O número de instares larvais foi determinado para as duas espécies: quatro para *Camponotus rufipes* Fabricius (Hymenoptera: Formicidae) e três para *Camponotus vittatus* Forel. De uma maneira geral, as larvas de machos das duas espécies se mostraram parecidas com as larvas anteriormente descritas no gênero, com as seguintes características em comum: formato geral do corpo e da mandíbula, presença da ‘chiloscleres’, ‘praesaepium’ (em alguns espécimes), pseudo—palpo labial e dez pares de espiráculos. Entretanto, as larvas das duas espécies somente puderam ser discriminadas através das medidas corporais, e diversidade e quantidade de pêlos. Comparando as larvas de operárias de *C*. *vittatus* previamente descritas com as larvas de machos, ambas são muito parecidas, podendo ser diferenciadas por características muito discretas, que podem ser devido a uma variação intraespecífica ou a uma variação entre os sexos.

## Introduction

The cosmopolitan ant genus *Camponotus* Mayr (Hymenoptera: Formicidae) comprises 1584 described species (Bolton et al. 2006), thus being a hyperdiverse group only to be rivaled by *Pheidole* (Wilson 2003). Some species of *Camponotus* are noteworthy as serious pests of wooden structures and bee nests (Akre and Hansen 1990), and also as common household pests, e.g., *Camponotus rufipes* Fabricius and *Camponotus vittatus* Forel in Brazil (Silva et al. 2009). In spite of the economic importance and diversity of *Camponotus* ants, there are few published studies on the morphology of their immature forms.

Schultz and Meier (1995) prepared a compared phylogenetic study using larvae of the tribe Attini, concluding that immature forms can provide good analytical characters; however, they are usually neglected during collection of insect samples, resulting in a general paucity of larval specimens in museum deposits. Wheeler and Wheeler (1953, 1968, 1970, 1974, 1991) prepared a series of pioneering larval descriptions with ants of several genera, including *Camponotus*. More recently, the immatures of workers of the species *Camponotus textor* (Solis et al. 2009) and *C*. *vittatus* (Solis et al. 2010b) were also described. Male ant larvae, which are more difficult to obtain, were seldom analyzed, and never were described within *Camponotus*. Adult male ants can often provide useful taxonomic and phylogenetic characters (Lapolla 2006; Yoshimura and Fisher 2011).

The present study thus aims to describe by light and electron microscopy the male larvae of the two species of *Camponotus*: *C*. *rufipes* and *C*. *vittatus*. The results are compared with previous descriptions with worker larvae to assess possible intersexual differences.

## Materials and Methods

### Collection of samples

Three nests of each species were obtained in the municipalities of Campinas (22^°^ 54′ 09.38″ S, 47^°^ 05′ 56.84″ W) and Rio Claro (22^°^ 23′ 44.09″ S, 47^°^ 32′ 39.98″ W), São Paulo, Brazil, and reared in the laboratory with a controlled room temperature of 23–27 ^°^C and 50–70% RH. In queenless colonies, some workers start laying eggs that only generate males. In the present study, the queens from the experimental colonies died; thus, only immature males were obtained for morphological description. Worker ants in queenless colonies are expected to lay eggs that eclose into male brood, and this phenomenon has already been observed in some species of *Camponotus* (Hölldobler and Wilson 1990). Specimens were fixed and conserved in 70% ethanol.

Voucher deposits of eggs, larvae, pupae, and adults were made in the “Adolph Hempel” entomological collection of Centro de Pesquisa e Desenvolvimento de Sanidade Vegetal of Instituto Biológico, São Paulo, Brazil.

### Immature descriptions

The number of larval instars was determined using the methods described in Parra and Haddad (1989), using 438 larvae of *C*. *rufipes* and 450 larvae of *C*. *vittatus*. 100 eggs, 175 larvae, and 30 pupae of *C*. *rufipes*, and 150 eggs, 109 larvae, and 30 pupae of *C*. *vittatus* were measured. Terminology follows Wheeler and Wheeler (1976). Samples were prepared and observed under light and electron microscopy as detailed in Solis et al. (2010b).

### Statistical analysis

All measured structures are presented below as minimal and maximal values, and measurements in tables are given in millimeters (mm). When comparing between the species, analysis of variance (ANOVA) was applied, and the differing figures were further compared by Tukey's test (α = 0.05). The following measurements were compared: length and width of eggs, first instar larvae, and last instar larvae. Measurements for worker larvae of *C*. *vittatus* were obtained from raw data from Solis et al. (2010b).

**Table 1.**
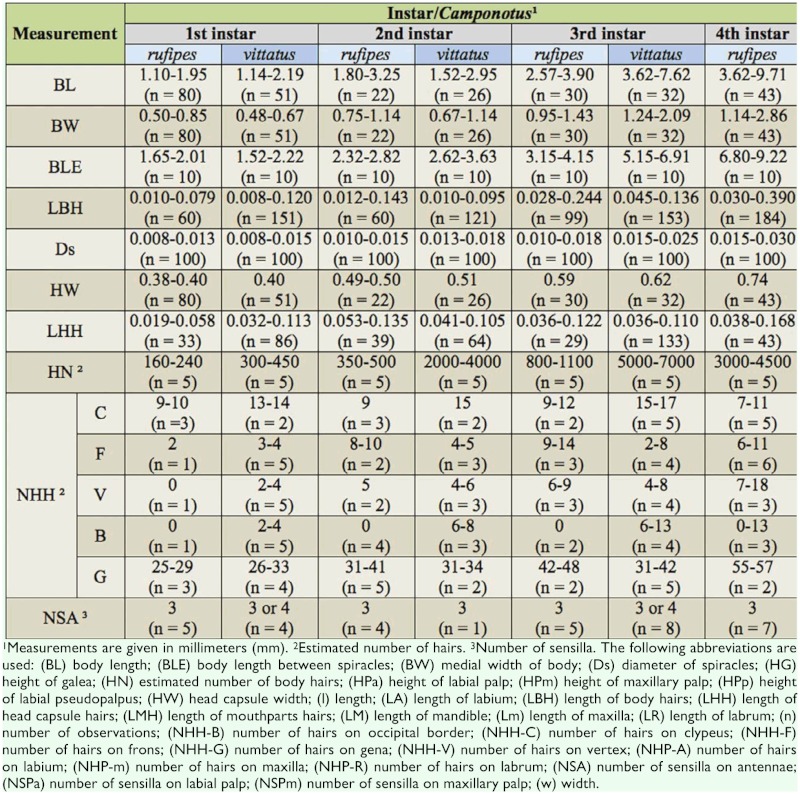
Body and head capsule of the male larvae of two species of *Camponotus*.

## Results

### Determination of number of larval instars

**Figure 1.**
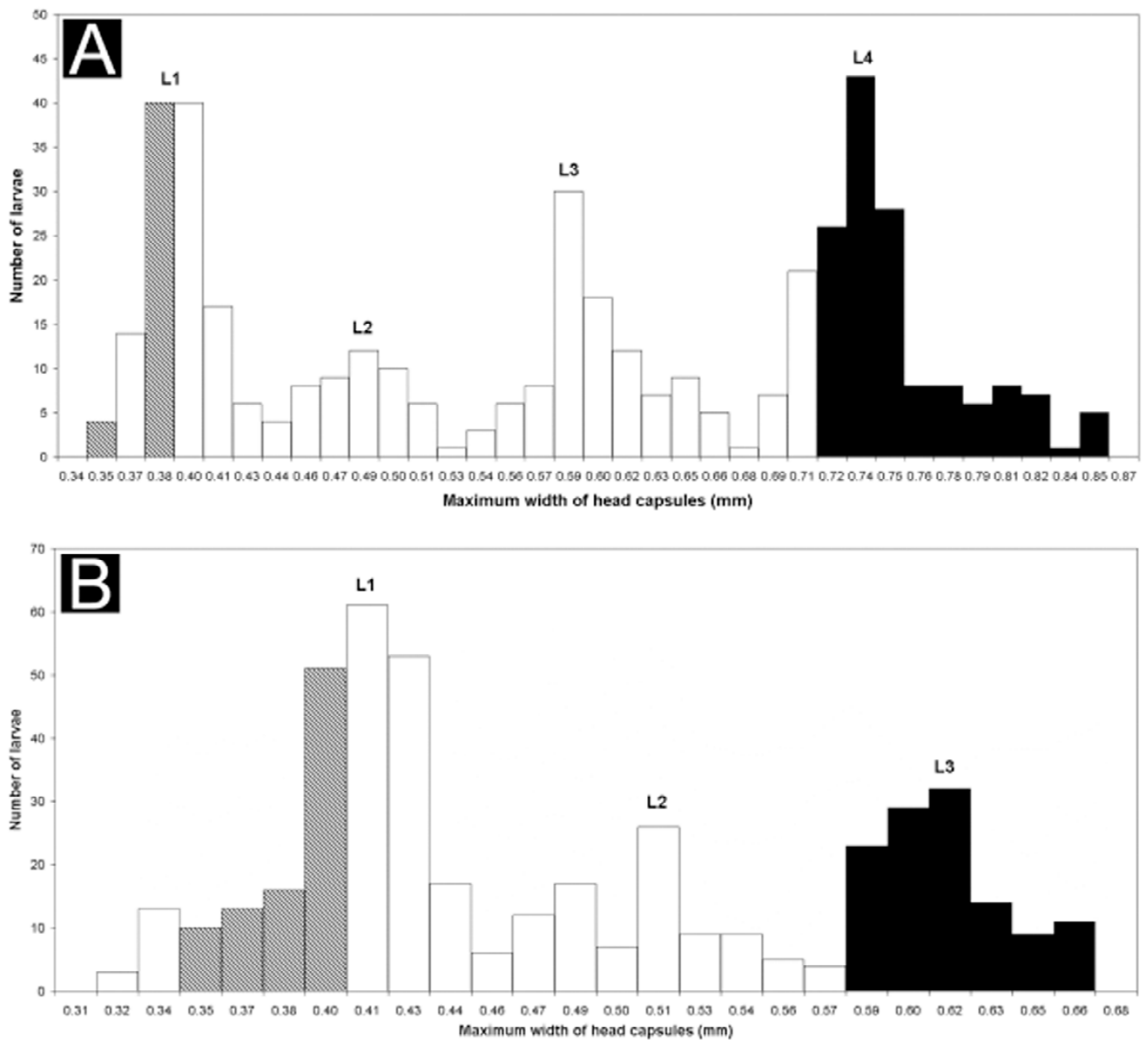
Frequency distribution of the maximum widths of head capsules of male larvae of *Camponotus* of different development stages: (A) *Camponotus rufipes*; (B) *Camponotus vittatus*. Abbreviations: (L1 ) first instar, (L2) second instar, (L3) third instar, and (L4) fourth instar. The hatched columns represent intervals in which mature embryos in the eggs were found. Black columns represent the interval in which prepupae were found. High quality figures are available online.

The frequency distribution of widths of larvae head capsules resulted in a multimodal distribution with four distinct peaks for *C*. *rufipes* and three for *C*. *vittatus*, suggesting these respective numbers of larval instars (Figure 1). In Solis et al. (2010b), the first peak represented first—instar larvae and the last peak prepupae. The obtained numbers of larval instars yielded a good fit with Dyar's rule (C. *rufipes*: R^2^ = 0.99; *C*. *vittatus*: R^2^ = 0.97).

Mean growth rate along the larval instars of *C*. *rufipes* was 1.24, with the rate from first—to— second = 1.23, second—to—third = 1.25, and third—to—fourth =1.25. Mean growth rate between larval instars of *C*. *vittatus* was 1.24, with the rate from first—to—second = 1.25, and second—to—third= 1.22.

### Morphological description of the immatures

Egg. Ovoid; *C*. *rufipes*: 1 = 0.88–1.79 mm, w =0.39–0.69 mm (n = 100); *C*. *vittatus*: 1 = 1.02– 1.39 mm, w = 0.47–0.63 mm (n = 150). Length:width ratio of *C*. *rufipes* 1.98; *C*. *vittatus* 2.13.

**Table 2.**
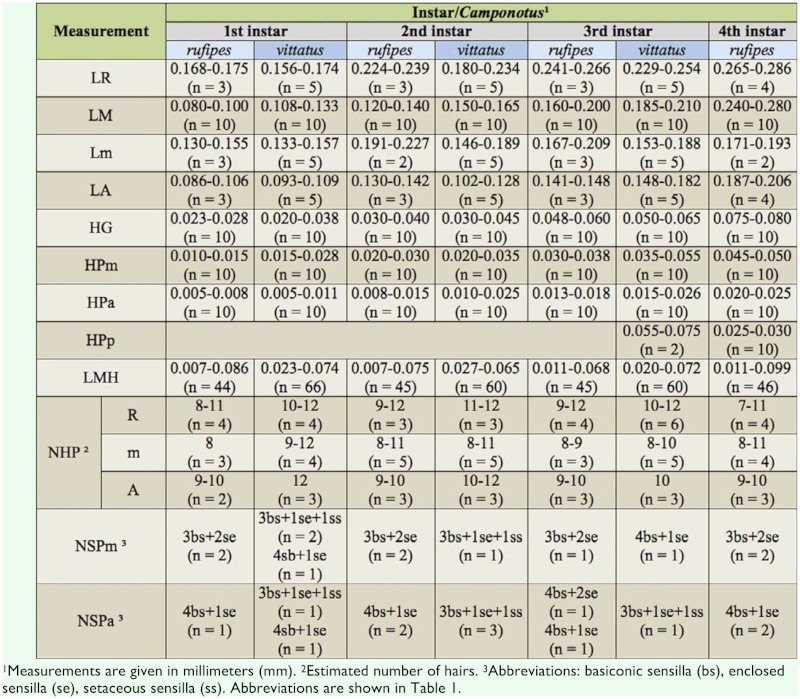
Mouthparts of male larvae of two species of *Camponotus*.

### General aspect of larvae

Male larvae of both species proved extensively similar to each other, and also to the worker larvae of *C*. *vittatus* described in Solis et al. (2010b). Thus, characteristics shared among these specimens are described below, and differences between are given in Tables 1–3.

**Figure 2.**
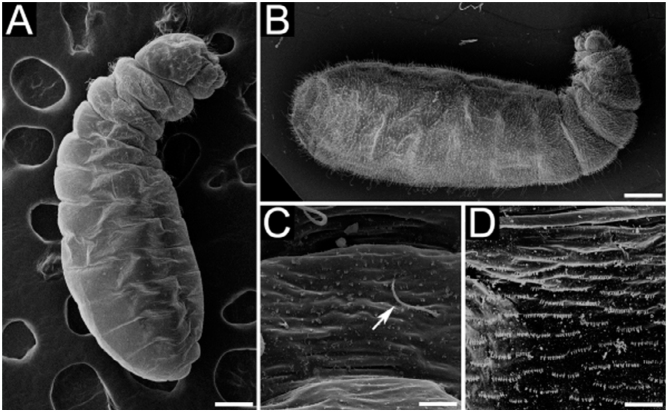
Scanning micrographs of male larvae of *Camponotus* on side view: (A) first instar of *Camponotus vittatus*; (B) fourth instar of *Camponotus rufipes*. Morphological aspects of the body of *C*. *rufipes* male larvae: (C) surface of the upper ventral integument of a first instar, showing rows of spinules and simple hair of subtype S1 (arrow); (D) surface of the upper ventral integument of a third instar, showing rows of spinules. Sizes of scale bars: (A) 0.160 mm; (B) 0.667 mm; (C) 0.018 mm; (D) 0.020 mm. High quality figures are available online.

Body shape pogonomyrmecoid (Figures 2A and 2B), anus subterminal. Body hairs abundant, yet scarcer upon ventral body surface, where there are rows of spinules (Figures 2C and 2D); spinules increase in size and abundance with every passing instar. Ten pairs of spiracles, first one slightly larger than the others, which are equally—sized. Head capsule subelliptical (Figure 4A) without spinules. Antennae with three basiconic sensilla (rarely four), which may or may not be arranged in line (Figures 4B and 4C). Clypeus clearly delimited from head capsule; gula with short spinules. Labrum subparabolic, with eight basiconic sensilla on ventral border (Figure 4F), and simple hairs on anterior face; posterior face covered with rows of spinules. Mandibles camponotoid, with striated surface (Figure 4D). Maxillae conoidal and elongate, bearing 8–12 hairs; maxillary palps with five sensilla; galea with two basiconic sensilla (Figure 4D); dorsum of maxilla with rows of spinules (Figure 4F), which increase in size after each molt. Labium rounded, with spinules in transversal rows above the opening of sericteries, which is a slit (Figure 4F); simple hairs on ventral border; labial palps usually with five sensilla (Figure 4E). Mature larvae have well—defined chiloscleres and labial pseudopalps, the latter with one basiconic sensillum on the side (Figure 4E).

**Table 3.**
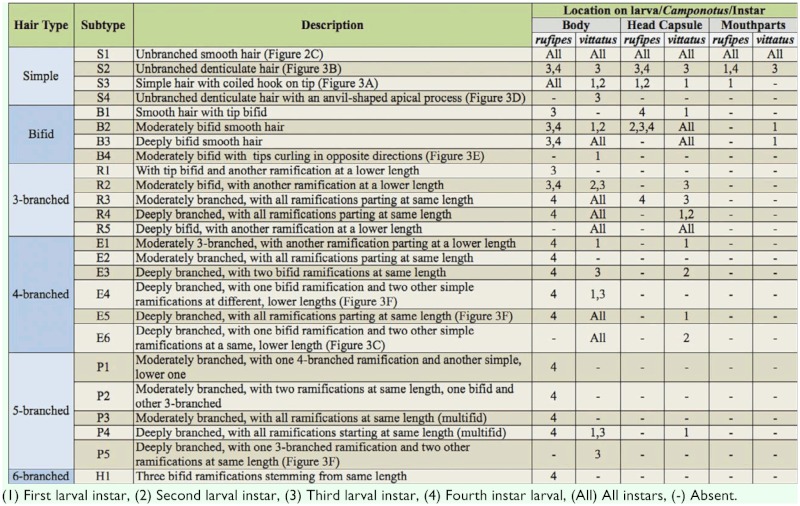
Types of body and head hairs found on larvae of Camponotus.

**Pupa**. Pupae exarate inside silky cocoons; meconium blackish, ejected inside the cocoon. Measurements of white pupae: *C*. *rufipes* body: 1 = 7.14–9.04 mm; head: w = 1.15–1.34 mm; (n = 30); *C*. *vittatus* body: 1 = 6.00–7.43 mm; head: w = 0.91–1.10 mm; (n = 30).

## Discussion

### Determination of number of larval instars

The recorded number of larval instars of ants varies between three and five, and this is the range recorded for species of *Camponotus* (Solis et al. 2010a). Few studies reported the number of larval instars in males. There are records of males with an additional larval instar (e.g. Arcila et al. (2002) with *Nylanderia fulva*), and also of males with the same number of larval instars (Masuko (1990) with *Amblyopone silvestrii*; Solis et al. (2010c) with *Linepithema humile*). In *Camponotus*, Bueno and Rossini (1986) found four instars for workers of *C*. *rufipes*, and Solis et al. (2010b) found three instars in C. *vittatus*. Thus, the present study reports that males of both species have the same number of larval instars as workers in the presented rearing conditions.

### Immature description

Hölldobler and Wilson (1990) mentioned the existence of two types of ant eggs: (1) trophic eggs that do not develop and are utilized as food, and (2) reproductive eggs that produce new individuals. Queens and workers are usually able to lay both types of eggs. According to these authors, in some species of the genera *Formica, Myrmica*, and *Pheidole*, the size of the reproductive eggs varies within females, with the larger eggs yielding queens, and the eggs of founding queens, usually smaller, originating minim workers. In the case of *Pheidole pallidula*, virgin queens can lay eggs of both types, with trophic eggs being larger (Passera 1978). Male eggs were of the same size as worker eggs in *C*. *vittatus* (figures compared with Solis et al. (2010b)), bearing in mind that the eggs laid by founding young queens were never measured. Eggs of *C*. *vittatus* proved slightly longer than male eggs of *C*. *rufipes*. One egg within our sample of *C*. *rufipes* was considerably different (67% longer above the mean and 26% narrower below the mean), and we think it could be a case of a trophic egg, yet as a single occurrence might also indicate malformation; no solid conclusion was reached.

**Table 4.**
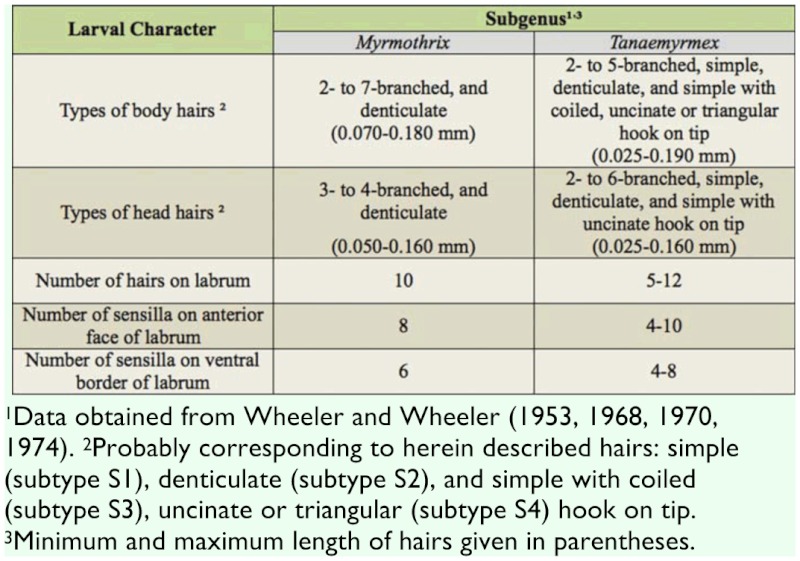
Differing larval characters of ant larvae from two subgenera within *Camponotus*.

Wheeler and Wheeler (1953, 1976) and Solis et al. (2009, 2010b) listed larval traits which are typical of *Camponotus* larvae, and they were confirmed in the present male specimens: body and mandible shape, presence of ‘chiloscleres’, ‘praesaepium’ (some specimens), and labial ‘pseudopalps’, and the existence of ten pairs of spiracles.

From comparing the two species, the male larvae of 1^st^ instar *C*. *vittatus* are longer and narrowed than those of *C*. *rufipes*, while male larvae are of the same size as workers within the same species. Regarding mature larvae, those of *C*. *vittatus* are smaller, with males also of the same size as workers. It is possible that the differences in size reflect the differences in size of the eggs and adults, e.g., adults of *C*. *rufipes* are slightly larger. Besides, it is possible that the existence of an additional instar would be necessary for the larvae to reach their ultimate size, considering that 3^rd^ instar larvae of *C*. *vittatus* are larger than those of *C*. *rufipes*. Valuable conclusions could be drawn from further comparison of the development durations of both species in future studies.

The two species analyzed in the present study belong to separate subgenera (*C*. *rufipes* in *Myrmothrix* and *C*. *vittatus* in *Tanaemyrmex*), and from comparing among previously described larvae of these subgenera (available at Wheeler and Wheeler 1953, 1968, 1970, 1974), it seems that the larvae from each subgenus differ only by the presence of one hair type (with hook on tip; see Table 4). This conclusion is at present preliminary, as there are few available described species for establishing a solid comparison (two *Myrmothrix* out of a total of 27; and 15 *Tanaemyrmex* out of 515); moreover, most descriptions employed smaller sample sizes without instar separation. For instance, male larvae of *C*. *rufipes* of all instars have hairs with a coiled hook on the tip. Additionally, the number of hairs in *C*. *rufipes* larvae increase in quantity and diversity of types with every subsequent instar, when hairs in *C*. *vittatus* only increase in quantity. Given any same instar, larvae of *C*. *vittatus* are always more hairy than larvae of *C*. *rufipes*. Regarding body hairs, as also verified with worker larvae of *C*. *textor* (Solis et al. 2009) and *C*. *vittatus* (Solis et al. 2010b), simple hairs of subtype S1 are present in all instars.

Male larvae of both species differed in the number of denticles on the mandible blade, with six in *C*. *rufipes* and seven in *C*. *vittatus*. However, this would not be of much use in species separation, as the workers of *C*. *vittatus* have six mandible blade denticles (Solis et al. 2010b).

From comparing the male larvae of *C*. *vittatus* with worker larvae of the same species described in Solis et al. (2010b), they proved morphologically similar in terms of size and shape of structures. However the following differences were noticed: maximum number of ramifications on body hairs (males: 5; workers: 6) and head hairs (males: 5; workers: 4); number of hairs on labrum (males: 10–12; workers: 8–11) and number of labrum sensilla (males: 8; workers: 12). Further comparisons using different nests would confirm if such differences can be used for sex discrimination, or if they are natural artifacts of intraspecific variation. The fact that male and worker larvae are similar is an exception to the observations of Wheeler and Wheeler (1976), who verified that larval morphology varied between individuals of different sex and castes, with the larvae of reproductive forms being larger when mature. Some species have even more conspicuous differences between male and worker larvae: Edwards (1991) noted that worker larvae of *Monomorium pharaonis* are covered with bifid hairs, while reproductive larvae are less hairy (with unbranched hairs) and greater in size; the author thought that maybe such differences would enable nursing workers to sort between both types of larvae. Solis et al. (2010c) noted that worker larvae of *L*. *humile* are slightly smaller than reproductive male larvae when mature, and present a dorsal protuberance upon the first abdominal somite; male larvae of this species lack this protuberance. Passera et al. (1995) verified that the workers of *L*. *humile* are capable of discerning the larval sex, age, and caste, probably based on chemical and morphological cues (possibly size and presence of a dorsal protuberance). As male and worker larvae of *C*. *vittatus* proved extensively similar, it is possible that only chemical signals are involved in sex discrimination in this species, or even that workers are not capable of larval sex discrimination. Nonacs and Carlin (1990) suggested that workers of *C*. *floridianus* are capable of detecting the sex of immature forms only upon pupal stage. This aspect deserves direct investigation. A case similar to the present study was reported by Masuko (1990) when dealing with larvae of *A*. *silvestrii*: separating larvae of different sexes was difficult, as the only observed difference was that male larvae were somewhat more hairy than female larvae.

Finally, the males of two *Camponotus* from the two different subgenera proved morphologically similar but with discrete, distinctive characters that may enable species and possibly sex—separation. The utility of such differences must be tested with numerous nests, and assessing their biological significance depends on further developmental data. Further descriptions of male larvae of *Camponotus* from other subgenera (including queenright and queenless males) are warranted to deepen general understanding of sex—related intraspecific variation.

**Figure 3.**
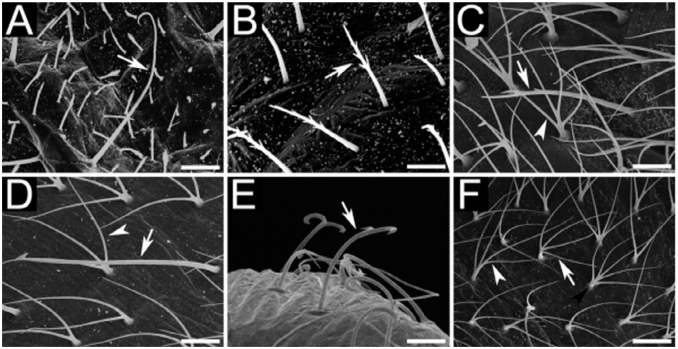
Types of hairs on the body of *Camponotus* male larvae: (A) subtype S3 (arrow); (B) subtype S2 (arrow); (C) subtypes S2 (arrow) and E6 (arrowhead); (D) subtypes S4 (arrow) and R4 (arrowhead); (E) subtype B4 (arrow); (F) E4 (white arrowhead), E5 (white arrow) and P5 (black arrowhead). Sizes of scale bars: (A) 0.077 mm; (B) 0.028 mm; (C) 0.016 mm; (D) 0.022 mm; (E) 0.015 mm; (F) 0.029 mm. High quality figures are available online.

**Figure 4.**
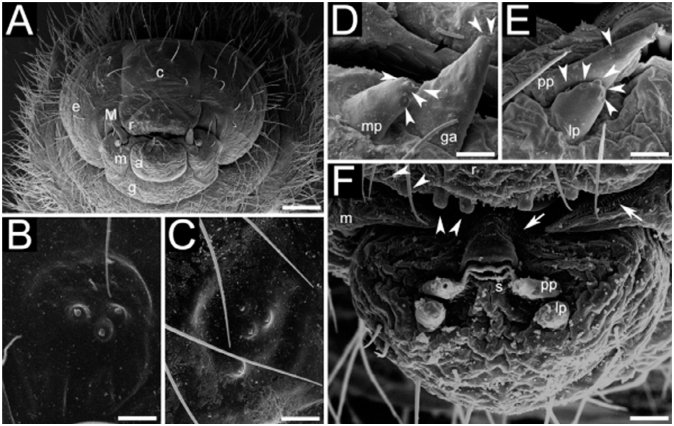
Scanning micrographs of head capsule and mouthparts of male larvae of *Camponotus*; (A) Head capsule of a fourth instar of *Camponotus rufipes*; (B) antenna of a second instar of *Camponotus vittatus*; (C) antenna of a third instar of *C*. *vittatus*; (D) details of mandibles and maxilla of third instar larva of *C*. *rufipes*; (E) pseudopalp and labial palp of fourth instar larva of *C*. *rufipes*; (F) labium of a fourth instar of *C*. *rufipes*. Abbreviations: clypeus (c), galea (ga), gena (e), gula (g), labium (a), labrum (r), mandible (M), maxilla (m), labial palp (lp), maxillary palp (mp), pseudopalp (pp), sensilla (white arrowheads), spinules (white arrow), striae (black arrowhead), sericteries (s). Sizes of scale bars: (A) 0.142 mm; (B) 0.010 mm; (C) 0.010 mm; (D) 0.013 mm; (E) 0.013 mm; (F) 0.020 mm. High quality figures are available online.
